# The Influence of Composite Thickness with or without Fibers on Fracture Resistance of Direct Restorations in Endodontically Treated Teeth

**Published:** 2014-07-05

**Authors:** Hassan Torabzadeh, Amir Ghassemi, Masoud Sanei, Sara Razmavar, Seyedeh Mahsa Sheikh-Al-Eslamian

**Affiliations:** a Iranian Center for Endodontic Research, Research Institute of Dental Sciences, Shahid Beheshti University of Medical Sciences, Tehran, Iran; b Dental Research Center, Research Institute of Dental Sciences, Shahid Beheshti University of Medical Sciences, Tehran, Iran; c Department of restorative Dentistry, Dental School, Shahed University, Tehran, Iran; d Dental faculty, Shahid Beheshti University of Medical Sciences, Tehran, Iran

**Keywords:** Composite Resins, Cusp Coverage, Cusp Reduction, Dental Restoration, Direct Restoration, Endodontically-Treated Teeth, Fiber, Stress Fractures, Resin Composite

## Abstract

**Introduction:** This *in vitro* study evaluated the influence of composite thickness (with or without fiber reinforcement) on fracture resistance of direct restorations in endodontically treated teeth. **Methods and Materials:** Fifty-six intact human premolars were chosen and randomly divided into four groups (*n*=14). After preparation of a mesio-occluso-distal (MOD) cavities and cusp reduction, the teeth were endodontically treated. Subsequently, the samples were restored with composite resin using the following protocols: group 1*;* composite onlay with cusp coverage of 1.5 mm, group 2*;* composite onlay with cusp coverage of 2.5 mm, group 3; composite onlay (including resin-impregnated fiber) with cusp coverage of 1.5 mm and group 4; composite onlay (including resin-impregnated fiber) with cusp coverage of 2.5 mm. The fracture resistance of teeth in all test groups was calculated by subjecting them to a progressively increasing compressive axial force in the universal testing machine with the cross-head speed of 1 mm/min to the point of fracture. The data were analyzed using the Kruskal-Wallis test. **Results:** The mean fracture strengths and obtained standard error were 1263.85±74.03 N, 1330.26±128.01 N, 1344.92±64.40 N and 1312.54±75.63 N for groups 1 to 4, respectively. Statistical analysis revealed no significant difference between groups. **Conclusion:** Cusp coverage of 1.5 and 2.5 mm in MOD access cavities with or without insertion of resin impregnated fiber had similar fracture rates in the endodontically treated teeth.

## Introduction

The amount of remaining tooth structure following access cavity preparation is one of the most important factors that influence the type and the technique of the final restoration of endodontically-treated teeth (ETT) [1], which are generally known to have reduced strength, principally due to the loss of tooth structure as a result of caries removal and access cavity preparation [2]. Accordingly, restoration of the root filled teeth remains one of the major concerns which should preserve an acceptable coronal seal as well as the tooth strength to increase its longevity [3].

Considering the cavity preparation procedures for amalgam or full coverage restorations, some state that these restorations may result in tooth structure being unnecessarily eliminated and thus weakened [4]. In addition, by advancements in mechanical properties of resin composites and due to their bonding ability to the tooth structure, along with their outstanding aesthetic properties, clinicians tend to use them in complex restorations, more specifically for ETT. Furthermore, the popularity of resin composites has increased due to their affordable expenses, reduced chair-side time, minimal technical sensitivity and reduced amount of tissue elimination during cavity preparation for direct restorations [5, 6].

**Figure 1:**
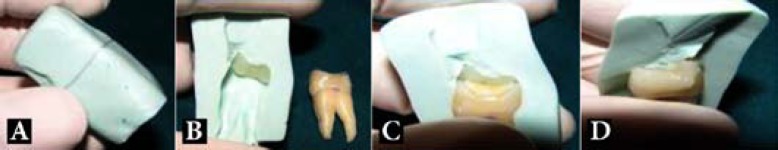
*A) *Silicon mould used for exact reconstruction of occlusal surface; *B-D)* Constructing the occlusal surface of restoration

Basically, it should be noted that compared to their extracoronal counterparts, intracoronal restorations put the ETT in a higher risk of fracture [7-11]. Thus, improvement in mechanical properties of adhesive materials can be considered effective in conservative tooth preparation. Furthermore, it is indicated that cusp coverage with adhesive materials is able to protect the cusps, and increase the tooth strength [12]. Regrettably, there is little evidence regarding the amount of cusp reduction which is desirable to promote tooth strength and preserve its appropriate function in the oral cavity. 

One of the methods that may affect the mechanical behavior of the teeth is the application of fiber within the composite; however, the effect of fiber on mechanical properties of the composites has been poorly investigated [13].

In order to substantiate the current information, this study was conducted to investigate the effect of composite thickness, with or without fiber, on the fracture resistance of direct restorations in ETT.

## Methods and Materials

In this *in vitro* study, fifty-six freshly extracted sound, non-carious human premolars were obtained according to the protocols approved by Ethical Committee of Dental Research Center, Shahid Beheshti University of Medical Sciences, Tehran, Iran.

Any calculus and soft tissue deposits were removed from the teeth using a hand scaler (Gracey Curette SG 17/18; Hu-Friedy, Chicago, IL, USA). Each tooth was carefully examined under light microscope (10×) for any existing enamel crack or fracture. Teeth were stored in 0.5% chloramine solution for 48 h, and then transferred to distilled water until the preparation time.

In order to minimize the effect of size and shape of the teeth on the results, the height and the width of each tooth was measured buccolingually and mesiodistally with a digital caliper (Mitutoyo CD15, Mitutoyo Co., Kawasaki, Japan) with an accuracy of 0.01 mm and then the teeth were classified according to their size obtained from the following equations:

Tooth height (H) = (Y1+Y2)/2, (Y1: distance between the buccal cusp edge from the CEJ; Y2: distance between the palatal cusp edge from the CEJ), tooth width (W)=width of teeth in height of contour area in palatal and buccal and then tooth size=H/W.

Thereafter, teeth in each size category were randomly distributed into four groups (*n*=14) as follows: group 1*;* restored with composite onlay with 1.5 mm cusp coverage, group 2; restored with composite onlay with 2.5 mm cusp coverage, group 3; restored with fiber-included composite onlay with 1.5 mm cusp coverage and group 4; restored with fiber-included composite onlay with 2.5 mm cusp coverage.

In order to have a precise reconstruction of occlusal surface in test groups, silicone moulds were prepared from each tooth using condensation putty heavy body (Speedex; Coltene, Altstatten, Switzerland) and then each mould was sectioned in occluso-apical direction and was used during occlusal reconstruction (Figure 1).

***Root canal treatment***

Mesio-occluso-distal (MOD) access cavities were prepared and root canal preparation was done by hand K-files (Mani, Tochigi, Japan) using step-back technique. The size of master apical file was 35 for all canals and they were obturated using lateral condensation of gutta-percha and AH-26 sealer (Dentsply, Tulsa Dental, Tulsa, OK, USA). During preparation, teeth were irrigated with distilled water to eliminate the effect of other irrigation solutions on their strength.

The MOD cavities were prepared with their buccal and palatal walls parallel to the buccal and palatal surfaces of each tooth. The gingival floor was placed 1 mm above the CEJ and the width of the cavities in the isthmus was two third of the intercuspal distance. Both buccal and lingual cusps were reduced 1.5 mm in groups 1 and 3 while in groups 2 and 4 the cusp reduction was 2.5 mm. The pattern of cusp reduction followed the cusp slope.

***Tooth restoration procedures***

The cavities were rinsed and etched with 37% phosphoric acid solution (Etchant Gel, Kerr Corporation, Orange, CA, USA) for 15 sec, and were then washed (30 sec) and dried (10 sec). Single bond (3M ESPE, St Paul, Minn., USA) was applied on the cavity walls according to the manufacture’s instruction and light cured with a quartz-tungsten-halogen (QTH) light curing unit (Arialux Blue Point, Ariadent, Tehran, Iran) for 20 sec. The cavities were then restored with Filtek Z250 (3M ESPE, St Paul, Minn., USA) using the incremental technique. The thickness of each layer was less than 2 mm which was light cured for 20 sec.

To construct the occlusal layer, a small amount of composite and the tooth was placed in the prepared moulds and was cured for 20 sec (Figure 2); finally finishing and polishing were carried out. In order to prevent desiccation of teeth, they were kept in distilled water during the test period. Unfilled-resin impregnated fiber (Fibrex Ribbon, Angelus Dental Solutions, Londrina, Brazil) was inserted into specimens in a buccal to lingual direction in groups 3 and 4 within the composite that was already placed in occlusal layer. The orientation of fiber followed the cusp slope and it was encompassed by at least 1 mm composite from each side. For this reason, first a thin layer of composite was placed on the occlusal surface and then it was covered by a piece of fiber prior to curing, then both composite and fiber were cured simultaneously for 20 sec, and finally, the last layer of composite was placed and cured as explained earlier.

**Table 1:** Mean, Standard Error (SE) and mode of failure of specimens

Mode of failure		Mean (SE)	Group (N)
Below CEJ (%)	Above CEJ (%)		
23	77	1263.85 (74.03)	1 (14)
70	40	1330.26 (128.01)	2 (14)
70	30	1344.92 (64.40)	3 (14)
45	55	1312.54 (75.63)	4 (14)

Teeth were stored in distilled water for 24 h and before the test, they were mounted in self-curing acrylic resin cylinders (Meliodent; Bayer UK Ltd., Newbury, UK) up to the cervical level 1 mm below the CEJ.

The cylinders were mounted in universal testing machine (Zwick/Roell, GmbH, Ulm, Germany) and were loaded with a cross-head speed of 1mm/min using a 4 mm diameter stainless steel rod which was attempted to be in touch with both buccal and palatal slopes in the midline between the tooth central fissure and the cusp edge. Loading process continued until tooth fracture.

At the end of the test, all the specimens were checked by naked eye, and then were divided into 2 groups according to the fracture position (*i.e*. above or below the CEJ). The mean and standard error (SE) of the results were calculated and statistical analysis was carried out using the Kruskal-Wallis and Chi-square tests.

## Results

The mean load (Mean±SE) for the tooth fracture in groups 1 to 4 was 1263.85±74.03 N, 1330.26±128.01 N, 1344.92±64.40 N and 1312.54±75.63 N, respectively. The highest and the lowest force required for fracture belonged to groups 3 and 1, respectively. However, statistical analysis revealed the absence of any significant difference between the groups (Table 1).

In the present study, the fracture was evaluated with naked eye and the position of fracture was considered according to the CEJ position. The fracture mode of the specimens is shown in Table 1. The Chi-square test did not show any significant differences between the groups (*P *value=0.236). According to the results, in groups 3 and 4 (teeth restored with resin composite containing fiber) the fracture mode was mainly above CEJ whereas the fracture pattern in groups 1 and 2 (teeth restored with resin composite without fiber) was below the CEJ level (Table 1).

## Discussion

This *in vitro *study investigated the effect of direct onlay restoration thickness with or without fiber on the fracture resistance of the ETT. Due to the fact that the shape, form and size of teeth have considerable effect on the fracture resistance, teeth were divided according to the crown length and tooth size since the dimensions of the prepared cavity depend on these factors.

The depth and the width of a cavity are the two major parameters in determining tooth resistance [14, 15]. It is reported that when the isthmus width is larger than one third of the intercuspal space, the cusp reduction should be taken into consideration; however, when the cavity width is extended to the two third of the intercuspal space, the cusp reduction becomes mandatory [15]. In the present study, the width of the MOD cavity in the isthmus was considered two-third of the intercuspal space, thus the cusp coverage of teeth was justified.

According to the previous studies, the ETT are more susceptible to fracture compared to the vital teeth; moreover, intracoronal restorations in the root-filled teeth are expected to expose them to a higher risk of fracture than extra-coronal restorations. Thus, reinforcement of the root-filled teeth against occlusal forces seems necessary [7, 16, 17]. In the current study, root canal therapy was carried out in all test groups.

The results in groups 1 (1.5 mm cuspal coverage) and 2 (2.5 mm cuspal coverage) revealed no significant difference in the fracture resistance of these two groups; thus, cusp reduction to an amount of 2.5 mm did not improve the fracture resistance of teeth more than 1.5 mm reduction. Accordingly, it can be hypothesized that a smaller cusp reduction (*i.e*. 1.5 mm) could probably preserve the tooth strength to the level of intact teeth without unnecessary tissue elimination.

The present study showed that there is no statistically significant difference in fracture strength of teeth with composite restoration compared to those restored with fiber-reinforced composite as an additional reinforcing strategy. This is probably due to the insufficiency of the fiber function within the composite. It is reported that the composition, form and direction of fibers, fiber/resin volume ratio and the bond strength between fibers and resin, have an influence on the reinforcing effect of fibers within the composites. Furthermore, they suggested that the mechanical property of the composite depends on type, extension and length of the fiber [3, 18].

It is indicated that when polyethylene fiber is applied within the flowable composite in the depth of the access cavity in ETT, the fracture strength of teeth is improved. This is probably due to the lower elastic modulus of the fiber-composite complex compared to the whole restoration that acts like a stress absorber and improves the fracture strength of the restoration [3].

On the contrary, Samadzadeh *et al*. [18] showed that the addition of 10% volume of plasma treated Ultra High Molecular Weight Poly Ethylene (UHMWPE) to the composite, reduced its flexural strength. Moreover, another study investigating the effect of fiber on poly (methyl methacrylate) composites, demonstrated that the fiber is not able to increase the fracture resistance of the whole complex. Deficiency in fiber-methacrylate bonding was suggested to be responsible for this result [18].

According to the study by Dyer *et al*. [19], fiber is able to improve the mechanical properties of the composite while the transmitted force directed from matrix to fiber. Also it was noted that there are some factors that may inhibit the proper bonding of fiber and resin, which demolishes the mechanical properties of the complex. One of the factors potentially responsible for the reduction of mechanical properties, is void formation in the matrix-fiber interface that may develope the unpolymerized and oxygen-inhibited layer within the structure of fiber reinforced composite (FRC) [19] and this can enhance the fracture tendency of composite through the matrix-fiber interface [20].

In the present study, the employment of the fiber within composites did not increase the fracture resistance of teeth. This is probably attributed to the existence of voids and defects in the body of the restoration. Although fiber was dipped into the unfilled resin prior to its application, the adaptation between fiber and composite was difficult to achieve due to the small size of the fiber, thus the defects were likely to increase. It is noteworthy to say that the defects and the voids not only make the restoration susceptible for mass fracture due to their weakening effect, but also expose them to water sorption through diffusion process. This could culminate in plasticization and weakening of the restoration [21]; however, further investigations are required to clarify the hypothesis.

The mode of fracture of a tooth could affect the type of restoration; therefore, in the present study the mode of fracture of teeth were also evaluated. According to the results, the predominant pattern of tooth fracture was below the CEJ level for groups 1 and 2 and above the CEJ for groups 3 and 4; hence, the application of fiber apparently affects the fracture pattern of the composite restorations. In this regard, Ellakawa *et al.* [22] investigated the fracture pattern of the fiber reinforced composites (FRCs). The results indicated that the fiber application led to the development of cracks which initially developed along the fiber orientation and then propagated to the body of the restoration [22]. It was also mentioned that the mode of fracture of a restoration depends on the fiber orientation [23].

In this case, the mechanical behavior of the material is strongly dependent on the load direction. In fact, while the load direction was almost perpendicular to the fiber direction, the behavior of a restoration is mostly influenced by the matrix and the filler rather than the fiber [24]. Moreover, in case of non-isotropic materials-as it is seen in teeth restored with fiber and composite-both flexural and shear loads exist together that lead to the production of diverse fracture patterns [19]. It is obvious that the adaptation of an *in vitro* test, in which the simple experimental specimens are restored with a non-isotropic material, to clinical conditions is complicated; therefore, the limitations of this study should be noted and mentioned as possible areas for further experimental researches mostly focused on limitation(s) of the *in vivo* environment.

## Conclusion

*i)* An amount of 1.5 mm cusp reduction is able to reinforce the teeth the same as reduction to the level of 2.5 mm.

*ii)* Fiber insertion within the composite restoration did not improve the fracture resistance of the teeth.

*iii)* Fiber insertion within composite was able to move the fracture level of the teeth to a restorable position.
